# Current Epigenetic Insights in Kidney Development

**DOI:** 10.3390/genes12081281

**Published:** 2021-08-21

**Authors:** Katrina Chan, Xiaogang Li

**Affiliations:** 1 Division of Nephrology and Hypertension, Mayo Clinic, Rochester, MN 55905, USA; katrinagracechan2021@gmail.com; 2 Department of Biochemistry and Molecular Biology, Mayo Clinic, Rochester, MN 55905, USA

**Keywords:** kidney development, epigenetics, DNA methylation, histone modification, single-cell RNA sequencing, single-cell epigenomics

## Abstract

The kidney is among the best characterized developing tissues, with the genes and signaling pathways that regulate embryonic and adult kidney patterning and development having been extensively identified. It is now widely understood that DNA methylation and histone modification patterns are imprinted during embryonic development and must be maintained in adult cells for appropriate gene transcription and phenotypic stability. A compelling question then is how these epigenetic mechanisms play a role in kidney development. In this review, we describe the major genes and pathways that have been linked to epigenetic mechanisms in kidney development. We also discuss recent applications of single-cell RNA sequencing (scRNA-seq) techniques in the study of kidney development. Additionally, we summarize the techniques of single-cell epigenomics, which can potentially be used to characterize epigenomes at single-cell resolution in embryonic and adult kidneys. The combination of scRNA-seq and single-cell epigenomics will help facilitate the further understanding of early cell lineage specification at the level of epigenetic modifications in embryonic and adult kidney development, which may also be used to investigate epigenetic mechanisms in kidney diseases.

## 1. Introduction

The kidney is one of the most complex organs in mammals and other vertebrates for maintaining homeostasis and filtering waste from the body, and it also produces and regulates several hormones and humoral factors, such as renin, erythropoietin, vitamin D, prostaglandins, insulin, gastrin, parathyroid hormone, thrombopoietin, glucose and urodilatin [1]. Each kidney is made up of about one million nephrons, comprised of more than 20 different specialized cells [2]. Each nephron consists of a glomerulus and a tubular system. The glomerulus filters the blood, and the tubule returns needed compounds and molecules to and removes waste from the blood. Nephrons are generated only during nephrogenesis, and *de novo* nephron formation continues until 36 weeks of gestation in humans [3]. The kidney has become the primary organ for studying the early embryonic mesenchyme and the signaling pathways that regulate its development and maturation into the mature kidney [4].

In humans, there are three stages of kidney development: pronephros, mesonephros and metanephros [5]. The pronephros is the earliest nephric stage in humans, and it is a vestigial structure that disappears completely by the fourth week of human embryonic life. The mesonephros develops by the formation of mesonephric tubules from the intermediate mesoderm, and it is the principal excretory organ during early embryonic life (4–8 weeks). The metanephros is derived from the intermediate mesoderm and arises caudal to the mesonephros at five weeks of development, and it is the permanent and functional kidney in higher vertebrates. [5] Both the pronephros and the mesonephros are primitive and exclusively embryonic stages, whereas the metanephros is the final stage that will become the mature adult kidney. However, the pronephros and the mesonephros still have important roles in the proper development of the metanephros [5]. Most of the kidney developmental processes involved in the proper differentiation of the various renal cell types are regulated by intricate genetic mechanisms [6]. Different signaling pathways and their roles in the proper development, differentiation and maturation of the different kidney stages and mature kidney structures have been identified [7]. Different gene expression during kidney development is also regulated by epigenetic mechanisms. This is essential for proper development through the various stages of kidney development and the continued maturation of the kidney postnatally [6].

Epigenetics is broadly defined as the heritable traits which are not directly encoded within the DNA of the genome. It is better defined as the heritable traits *through mitosis or meiosis* which are not directly encoded within the DNA of the genome. The field of epigenetics developed from research aimed at understanding the mechanisms by which multiple cellular phenotypes may arise from a single genotype [8]. The term “epigenetics” was originally limited to mechanisms that could retain the phenotypic state (differential gene expression) through cell division via non-genetic factors, to avoid confusion with such changes associated with environmental factors, such as nutrition and stress [9]. This includes enzymatic methylation of cytosine bases or DNA methylation, post-translational modification of tail domains of histone proteins or histone modification, associated nucleosome positioning or chromatin remodeling, the effects of non-coding RNAs and the roles of transcription factor regulatory networks [10].

Epigenetics influencing gene expression can be divided into intragenerational and transgenerational epigenetics [8]. Intragenerational epigenetics involves the modification of gene expression through epigenetic marks that result in a modified phenotype within an individual’s lifespan. These mainly include DNA methylation, covalent histone modification and microRNA actions [11]. Transgenerational epigenetics is defined as the inheritance of a modified phenotype from the parental generation without changes to the genomic sequence [11]. It is important to note that the two types of epigenetics are not mutually exclusive. These epigenetics pathways are important to the proper timing of the differentiation of the different renal cell types, as well as the regulation of these differentiating structures. Because of the regulatory role of the epigenetic mechanisms, the closer evaluation of these mechanisms has led to new discoveries about organ development and the progression of disease such as cancers and to novel approaches to treating these diseases [12]. With a growing body of literature on epigenetics, this review focuses on the genetic and epigenetic mechanisms that regulate kidney developmental stages and the single-cell sequencing techniques used in studying these mechanisms.

## 2. Overview of Epigenetic Mechanisms

The main mechanisms of epigenetics involve DNA methylation, histone modifications and miRNA activities [13]. DNA methylation usually occurs in the form of 5-methylcytosine (5MC) events, which is the most stable epigenetic marker and plays a significant role in gene regulation and heterochromatin maintenance [14]. This is hugely important, because this type of methylation can be found in abundance in the genome, particularly in CpG island protomer regions [15]. A lack of methylation in the majority of CpG islands in the promoter regions of genes allows for the genes to be transcribed. Hypermethylated CpG islands usually occur in inactivated X-chromosomes, imprinted genomic regions and improperly silenced genes [16]. DNA methylation is considered to be an active process and is reversible, which is accomplished by specific DNA demethylases, such as the TET family [17]. This reversibility has a role in embryonic development and in DNA damage repair.

Histone modifications largely involve methylation and acetylation events. In all mammalian nuclei, DNA bundles together, forming chromatin, which contains a protein core of histone protein dimers (H2A, H2B, H3 and H4) wrapped by approximately 150 bp sections of double-stranded DNA [18]. Histone tails are the primary location for modifications, particularly lysine residues, and they serve as a roadmap of specific post-translational modifications of regulatory elements within the genome [18]. Different modifications may result in a closed (transcriptionally silent) or open (transcriptionally active) conformation, which allows certain genes to be expressed spatially and temporally. Therefore, chromatin has two subtypes: heterochromatin, which is transcriptionally silent, and euchromatin, which is transcriptionally active [18].

Histone methylation and acetylation are the most common histone modifications that may help regulate gene expression. These usually occur on lysine and arginine residues. Histone methyltransferases (HMTs) regulate histone methylation, which for a long time was thought to be irreversible, but histone demethylases have since been characterized [19]. Some suppressors of variegation 3–9 (Su(var)3–9)/Enhance of Zeste (E(z))/trithorax (Trx) (SET) domain-containing proteins have HMT activity and have catalytic activity towards lysine resides for mono-, di- or trimethylations by interacting with the methyl donor S-adenosyl methionine (SAM) [20]. Mono-, di- and trimethylation can occur on the same lysine of histones to either promote or repress gene transcription [11]. For example, trimethylation of lysine 4 residues of histone H3 (H3K4me3) at transcription start sites promotes gene transcription, and di- or tri-methylation of lysine 9 of histone H3 (H3K9me2/3) and tri-methylation of lysine 27 of histone H3(H3K27me3) repress gene transcription [21]. Arginine methylations also play a role in gene regulation and can occur singularly (s), double symmetrically (2s) or double asymmetrically (2as) [21]. In general, histone arginine H3R2 double symmetrical methylation (H3R2me2s) is repressive, histone arginine H3R17 double asymmetrical methylation (H3R17me2as) is active and histone arginine H3R2 double symmetrical methylation (H3R8me2s) can be either repressive or active [21].

Acetylation of certain histone dimers plays a role in regulation of the opening of the associated chromatin. Histone acetyltransferases (HATs) facilitate the acetylation of histones by neutralizing positive charges of lysines and consequently decreasing histone–DNA interactions [11]. Conversely, histone deacetylases (HDACs) have a role in gene repression, and the combination of these processes help regulate transcription and subsequent gene expression [22].

Other post-translational histone modifications include histone ubiquitination, sumoylation and ADP ribosylation [11]. Histone ubiquitination may have a role in transcriptional regulation and cellular response to DNA damage [23]. Histone sumoylation, in which a small ubiquitin-related modifier (SUMO) protein is added onto a histone in a similar manner to protein ubiquitination, may modulate gene expression [24]. Histones can be ADP-ribosylated in Asp/Glu residues by ADP-ribose transferases (ARTs), which play an important role in DNA damage repair [25]. Certain histone variants, such as H2A, H2AX and H2AZ variants of histones, have a role in DNA repair [26]. Phosphorylated H2AX (γH2AX) identifies double-stranded breaks and helps initiate DNA repair mechanisms. Because of their regulatory functions, several mutated forms of histones have been associated with cancers and are known as oncohistones.

Another group of epigenetic mechanisms has arisen from epitranscriptomics, or the study of small non-coding RNAs (ncRNAs) and their roles in the regulation of gene expression. These ncRNAs can regulate protein levels and are themselves regulated by epigenetic modifications [27]. This has led to the novel usage of siRNAs (small interfering RNAs) to target and destroy its complementary mRNA and effectively silence a gene. RNAi-defective 1 (RDE1) and Dicer are vital for the RNA interference (RNAi) process, in which an RNA-induced silencing complex (RISC) is guided by siRNA to the target mRNA and destroys it [28]. Long non-coding RNAs (LncRNAs) also have a significant role in genetic expression [29]. In particular, Xist regulates X inactivation, where one of the X chromosomes in a female is silenced, creating inactive Barr bodies [30].

## 3. Overview of Human Kidney Development

Embryonic development is a highly complex process involving many important signaling pathways that initiate the different stages of organogenesis. Human kidney development begins after the development of cardiovascular and nervous systems in the first several weeks of embryogenesis [31], which becomes evident at day 22 in week five of gestation, with the appearance of the Wolffian duct, or more commonly known as the nephric duct [5]. This duct is derived from the mesoderm, the middle of the three germ layers (ectoderm, mesoderm and endoderm) from which most major organ systems develop. The mesoderm has three regions, including the paraxial mesoderm, the intermediate mesoderm and the lateral plate mesoderm. Embryonic kidneys arise from the intermediate mesoderm [32].

**Figure 1:**
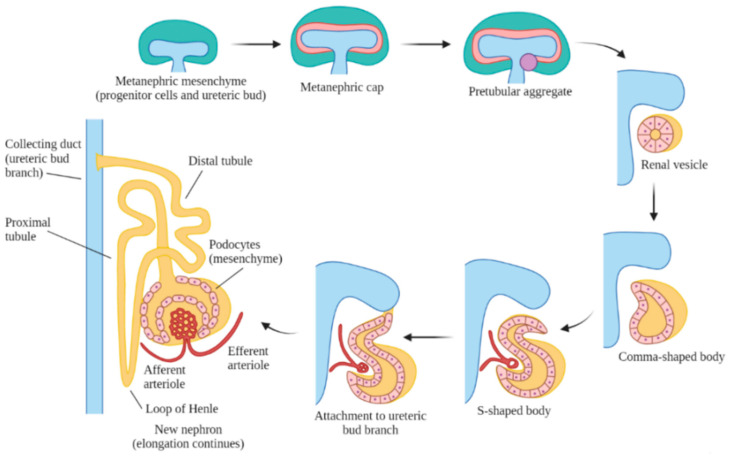
Formation of nephrons in the metanephros. The ureteric bud signals for the metanephric mesenchyme to form a cap around it, which signals the formation of the renal vesicle. The vesicle elongates into the comma-shaped, then the S-shaped body, before attaching to the ureteric bud branch and further differentiating into a nephron. The nephron continues to elongate and mature throughout the prenatal period.

Embryonic kidney development includes three stages: pronephros, mesonephros and metanephros [5]. The pronephros appears in week five but disappears within a week. This is a primitive, non-functional kidney, consisting of vestigial nephrons. The mesonephros is functional for only a short period of time, and it remains as the mesonephric (Wolffian) duct, which is generated from the pronephric duct in week six. The metanephros is the final kidney stage and remains as the permanent adult kidney, which appears in week seven as a bud from the caudal end of the mesonephric duct, also known as the ureteric bud. The ureteric bud branches and develops into the ureter, renal pelvis, calyces and the collecting tubules. The ureteric bud is responsible for the majority of cell signaling in nephron endowment and formation [33]. During this process, the ureteric bud grows into the intermediate mesoderm and signals nearby cells to form a metanephric cap onto the bud, which forms nephrons that connect to the collecting tubules (Figure 1).

In the metanephros, the nephron and associated structural development occur over four periods [5]. Period 1 takes place during embryonic weeks 5–14, during which the ureteric bud is actively branching into new buds, and the associated metanephric cap actively forms new nephrons to newly forming ureteric bud branches. Period 2 takes place during embryonic weeks 14/15–20/22, during which the ureteric bud forms few new bud branches but induces new nephrons to form into a chain of nephrons connected to each bud branch. Period 3 takes place during embryonic weeks 20/22–32/36, during which the ureteric bud stops forming new bud branches and only induces formation of new nephrons to the remaining new bud branches and behind the active growth zones where collecting tubules have formed. Finally, period 4 occurs during embryonic weeks 32/36–adulthood. during which no new nephrons or budding branches are formed. However, renal growth in this period consists of existing structures increasing in size and length and the expansion of the surrounding tissues.

The majority of nephron maturation occurs postnatally when the formation of new nephrons has stopped. Human kidneys have approximately one million nephrons at birth [34]. In matured nephrons, there is a renal corpuscle, containing the Bowman’s capsule that is connected to the distal and proximal tubular system and finally drains into the collecting duct [35]. Nephrons are supplied by the complex network of the renal vasculature, which is connected to the main circulatory system [36]. The nephrons are organized in renal pyramids, and the bases originate at the corticomedullary border, and the apex terminates at a papilla within a minor calyx. Minor calyces collect urine from the papillae and expand into the major calyces, which extend into the renal pelvis, which then extends into the ureter [37]. There are two types of nephrons based on the location of the renal corpuscle, superficial and juxtaglomerular nephrons [38]. Superficial nephrons do not extend past the outer medulla and are not supplied by a vasa recta, whereas juxtaglomerular nephrons extend into the inner medulla and are supplied by a vasa recta [38]. The tubular part of a nephron consists of the proximal tubule connected to the distal tubule by the loop of Henle. The Bowman’s capsule, containing the glomerulus, sits between the efferent and afferent arterioles. The macula densa is a short segment of the distal tubule that sits at the intersection between the distal tubule and the glomerular tuft [39].

The exact mechanisms of the formation of the renal vasculature are not well understood, but its developmental patterns and structural presentations have been studied [5]. The first signs of vasculature appear during weeks 6–8 of embryonic development. During this early stage, the S-shaped bodies begin developing glomerular capillary loops, and consequently, the complex renal vasculature begins to develop. It remains unknown if the renal vasculature results from angiogenesis or vasculogenesis. Angiogenesis involves proliferation from existing endothelial cells and vascular cells, most likely from vessels originating from the aorta [40]. Vasculogenesis involves *de novo* formation of blood vessels from the endothelial mesenchyme [40]. During the S-shaped body stage, glomerular capillary loops begin developing, and consequently, the renal vasculature begins developing. The renal artery branches into a network of arteries, ending with the afferent arteriole, which expands into the glomerular capillaries, which then aggregate to form the efferent arteriole. The efferent arteriole then branches into the peritubular capillaries, which supply blood to the kidneys. In a similar organizational pattern, the renal venous system runs parallel to the arterial system and consists of the interlobular vein, the arcuate vein, the interlobar vein and the renal vein. This complex vasculature develops synchronously with nephrogenesis and is vital to proper kidney development and function throughout adulthood [36].

## 4. Major Genetic Markers and Signaling Pathways in Kidney Development

**Figure 2:**
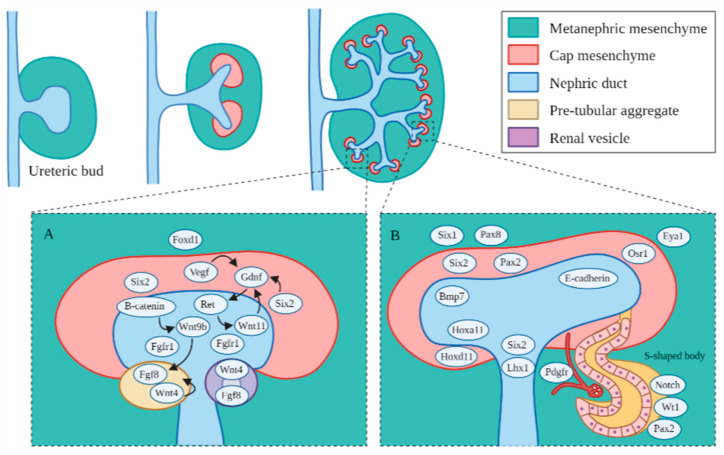
Major genes and signaling pathways in the formation of the ureteric bud branches, mesenchyme cap and subsequent nephrons. (**A**) Regulatory signaling pathways identified in the early metanephric mesenchyme. *Foxd1* regulates specification of the metanephric mesenchyme to form the ureteric bud cap. *Vegf*, *Six2* and *Wnt11* are vital early progenitor factors that activate the *Gdnf/Ret* pathway for the proper branching of the ureteric bud and subsequent nephron formation. *Fgf1* also contributes to proper ureteric bud branching in the ureteric bud. *Fgf20* regulates *Fgf1/2* in the formation of the ureteric cap. *β-catenin* mediated the induction of *Wnt9* regulates *Wnt4* and *Fgf8*, which are critical for renal vesicle formation. (**B**) Key genetic markers identified in the metanephric mesenchyme and nascent nephrons. *Hoxa11* and *Hoxd11* regulate ureteric bud growth. *Six1* and *Six2* are important for continued mesenchyme differentiation. *Pax2* and *Pax8* are important for continued nephric duct formation. *E-cadherin* and the other cadherins indicate the segmentation of the S-shaped body, and *E-cadherin* is expressed in the distal segments where the S-shaped body joins the ureteric bud. *Pdfgr* plays a role in the formation of the glomerulus.

The development and differentiation of the intermediate mesoderm are dependent on complex signaling pathways [6] and are shown in Figure 2. Several genetic markers or transcriptional regulators, including homeobox (*Hox*) paralogs, odd skipped related 1 (*Osr1*), Paired box protein 2/8 (*Pax2/8*) and eyes absent 1 (*Eya1*), play major roles in early patterning and specification of the developing kidney [6]. *Hox* genes function in regional specification and patterning of the axial skeleton and the central and peripheral nervous systems. Redundancy is evident among the paralogous groups within the four groups of *Hox* genes: *HoxA*, *HoxB*, *HoxC* and *HoxD*, which play important roles in various regions of the developing embryo [41]. Of all of the *Hox* genes, *Hox 11*, consisting of *Hoxa11*, *Hoxc11* and *Hoxd11*, plays a critical role in kidney development. In particular, *Hoxa11* and *Hoxd11* play a role in the branching of the ureteric bud and subsequent metanephric growth [41]. The Lim type homeobox gene (*Lhx1*) is present in the lateral plate mesoderm and serves as a marker of the developing posterior and lateral mesoderm [42]. *Lhx1* becomes more restricted to the intermediate mesoderm and to the nephric duct as it begins to develop and promotes the continuing growth of the nephric duct [42]. *Osr1* encodes the zinc-finger DNA binding protein, one of the first markers of the lateral plate mesoderm and intermediate mesoderm [42]. *Osr1* continues to be expressed by metanephric progenitor cells but is not expressed in *Pax2*-expressing cells of the growing nephric duct, and it may have a role in promoting proper differentiation of the metanephric mesenchyme from the posterior intermediate mesoderm [42]. Redundancy between *Osr1* and *Osr2* may contribute to continued expression of *Pax2* with only one or the other [43]. *Pax2* and *Pax8* are markers only found in the intermediate mesoderm, which promote proper formation of the nephric duct [44]. *Hox* gene expression patterns may regulate how the mesoderm responds to intermediate mesoderm differentiation signals, which in turn, may initiate the expression of *Lhx1*, *Pax2* and *Pax8* along the posterior axis of the developing embryo [6]. *Hox11* regulates the glial cell-line-derived neurotrophic factor (*Gdnf*) and sina oculis-related homeobox 2 (*Six2*) expression, which further regulates the differentiation of the metanephric mesenchyme from the mesonephric tissue and contributes to the initiation of the proper development of the metanephros [45]. The expression of *Eya1* and *Pax2* is necessary for *Six2* gene activation in the metanephric mesenchyme [46]. Wilms’ tumor suppressor (*wt1*) is expressed all along the anterior–posterior axis within the intermediate mesoderm and is associated with Wilms’ tumor when it is incorrectly regulated [47]. Activin and retinoic acid are known to promote intermediate mesoderm marker gene expression and renal development [48]. Activin induces *Lhx1* expression and may interact with other signals from the neural tube and the ectoderm to regulate the mediolateral positioning of the metanephros. Additionally, bone morphogenetic proteins (BMPs) activate intermediate mesoderm- and lateral mesoderm-specific genes [49].

Branching morphogenesis is tightly regulated by different growth factors. such as GDNF [50], vascular endothelial growth factor (VEGF) [51] and fibroblast growth factors (Fgfs) [52]. GDNF and VEGF are secreted from the metanephric mesenchyme, and they interact with each other in regulating ureteric bud branching [53]. Fgf7/10 plays a role in the development of the collecting ducts [52]. Fgf8 induces the formation of the metanephric caps and may regulate *Wnt4* and *Lhx1* expression. Fgf9 and Fgf20 are secreted by ureteric bud, which can maintain proper cap progenitor cell proliferation [52]. Fgfs and Bmp7 provide survival signals for the metanephric mesenchyme, metanephric cap progenitor cells and may have a role in the growth of stromal cells that support the metanephric cap progenitor cell density [54]. Binding of these growth factors to their tyrosine kinase receptors activates three major signaling pathways: RAS/mitogen-activated protein kinase (RAS/MAPK), diacylglycerol protein kinase C/mitogen-activated protein kinase (DAG/PKC/MAPK) and phosphatidylinositol 3-kinase/protein kinase B (PI3-K/AKT) pathways [55]. These pathways play important roles in mitotic proliferation, survival and migration of ureteric bud cells.

In the ureteric bud and collecting ducts, RET (receptor tyrosine kinase), GDNF and its co-receptor, GDNF family receptor α 1 (GFRα1), initiate a signaling cascade that triggers the growth of RET-positive cells from the nephric duct towards GDNF cells of the metanephric mesenchyme [50]. A network of inhibitors regulates GDNF/RET signaling to prevent improper ureteric bud branching. BMP4, a member of the TGF-β super-family, inhibits excessive GDNF/RET signaling in the metanephric mesenchyme, which can be blocked by the BMP antagonist gremlin (Grem1) [56]. VEGF can promote phosphorylation of RET to regulate ureteric bud and glomerular development [53]. Sprouty homolog 1 (*Spry1*) also regulates RET signaling [57]. There are several other genes that regulate ureteric bud formation and development. Slit homolog 2 (*Slit2*) and its receptor roundabout homolog 2 (*Robo2*) regulate the anterior intermediate mesoderm and prevent improper ureteric bud branching [58]. Phosphatase and tensin homolog (PTEN) also regulate proper ureteric bud growth [59]. Fibroblast growth factor receptor 2 (Fgfr2) regulates ureteric bud branching and nephron endowment [60]. *Eya1* interacts with *Six1* in regulating the differentiation of the metanephric mesenchyme in early development [61].

In the metanephric mesenchyme, *Wnt9b* encodes the only WNT protein that induces formation of the metanephric mesenchyme, with β-catenin sometimes mimicking *Wnt9b* activity as a redundant gene signaling pathway in the occurrence of the early absence of *Wnt9b* [62]. *Foxd1* (retinoic acid receptors) has a role in regulating ureteric bud branching [63]. Transcription factor 21 (*tcf21/pod1*) is expressed by stromal cells and is necessary for continued epithelial cell production [64]. Most of the metanephric stromal cells come from the paraxial mesoderm, and few stromal cells come from the intermediate mesoderm after the metanephric ureteric bud has formed. The Notch pathway (ligands Delta, Jagged and Serrate) are known to regulate differentiation of renal cell types and may bypass Wnt signaling to induce the development of the metanephric mesenchyme and subsequent differentiation into renal epithelial cells [65].

During the nephron formation process, *Cited1* or *Six2* regulate the differentiation of the metanephric caps into glomerular, proximal tubular and distal tubular epithelia [66]. *Six2* plays a role in the formation of renal vesicles, the most primitive and first recognized nephron structure forming from the enlarged pretubular aggregates, and which subsequently elongate through several phases, including the comma and S-shaped bodies, to become nascent nephrons (Figure 2) [67]. The metanephric cap formation requires the *β-catenin*-mediated signaling of *Wnt9b* and *Wnt4* to properly form renal vesicles [68]. In the patterning of the nephron, differential expression of different cadherin genes has a role in the differentiation of various segments of the renal vesicle into the glomerular (P-cadherin), proximal (cadherin-6) and distal segments (E-cadherin) [69]. The distal segments fuse to the collecting ducts and integrate the distal vesicle cells into the appropriate collecting tubules, and this arrangement becomes apparent by the S-shaped body stage. During this patterning, the VEGFs signal the formation of the glomerulus, and it becomes evident by morphological changes in the podocytes towards the mature glomerular epithelium [70].

## 5. Epigenetics in Embryonic Development

The blastocyst stage before implantation into the uterine wall is characterized by unique molecular and morphological changes [12]. The differentiation from the fertilized egg to the zygote to the embryo requires multiple epigenetic signaling pathways to occur at the right time for proper development. One of the most important steps is that the zygotic epigenome undergoes a complete restructuration via DNA demethylation and a thorough remodeling of histone post-translational modifications [71]. During the fetal period, tissue differentiation, organogenesis and fetal growth are under the control and protection of the placenta, and therefore, maternal metabolic diseases can affect fetal growth and are often associated with DNA methylation changes [72]. In the postnatal period, the maturation of several organs, including the intestines, liver, adipose tissue and nervous system organs, are also regulated by epigenetic mechanisms [73]. Interestingly, breastfeeding may be associated with changes in DNA methylation on the promoters of leptin (*LEP*) and neuropeptide Y (*Npy*), which encode anorexigenic and orexigenic hormones [74]. Additionally, long-term environmental effects may result in epigenetic changes, and these epigenetic marks can be transmitted through cell generations and are preserved during mitosis, which can lead to altered gene expression and increase the risk of disease during adulthood [75].

## 6. Major Epigenetic Markers and Epigenetic-Mediated Signaling Pathways in Kidney Development

Epigenetic mechanisms have been found in the regulation of kidney development [76]. Throughout nephrogenesis, DNA methylation and histone modification events regulate the proper differentiation and formation of the kidney structures. Because chromatin structure and gene-specific histone modifications cannot be observed directly in the embryo, and gene expression patterns cannot be compared easily, finding and dissecting the epigenetic mechanisms has not been without challenges [77]. However, significant progress has been made in the past decade.

During kidney development, DNA methylation is catalyzed by DNA methyltransferase 1 (DNMT1), DNMT3a and DNMT3b [78]. DNMT3a/b are essential for *de novo* DNA methylation during early prenatal development, just shortly after implantation, and during subsequent embryonic development. DNMT1 functions to maintain DNA methylation in the mammalian genome for proper development of the kidney [78]. DNMT1 and DNMT3a is highly expressed in the nephrogenic mesenchyme, including the ureteric bud, renal vesicles and the comma and S-shaped bodies, whereas DNMT3b is only weakly expressed, and all three DNMTs’ expression is significantly reduced in maturing and adult kidneys [79]. DNMT1 plays an important role in the maturation of kidney structures, proper kidney growth, nephron formation and endowment and self-renewal of the renal progenitor cells [78]. The expression of DNMT1 has been associated with the proper expression of *Cited1*, *Hoxd11*, *Hoxd8*, *Wt1*, *Six2*, *Eya1* and *Osr1,* as well as ligands of the Wnt signaling pathways [78]. *Wt1* and *Sox11* are implicated in gene expression, particularly of the *Wnt4* genes, and allow for the proper differentiation of the metanephric mesenchyme into epithelial cells during tubulogenesis [80]. In the absence of DNMT1, these genes are downregulated in varying degrees, suggesting a secondary gene downregulation as a result of the intermediate gene dysregulation [78]. Because of its multiplex functions, DNMT1 is associated with the proper regulation of the progenitor cell network and with the overall proper differentiation of these cells into the appropriate kidney structures, particularly structures derived from the cap mesenchyme [78].

Histone modification also plays an important role in the regulation of kidney development. The levels of H3K9me2 and H3K27me3 are elevated in *Six2*-expressing nephron progenitor cells, resulting in repressing gene transcription until differentiation is triggered [81]. Once triggered, the levels of H3K4 tri-methylation are increased, and the levels of H3K9 di- and tri-methylation and H3K27 tri-methylation are decreased in those cells, and subsequently, *Pax2* and *Lhx1* are activated, and differentiation of the cap mesenchyme into new ureteric bud branches and nascent nephrons can be initiated [21]. Histone lysine methylation of activating H3K4 and repressive H3K27 also occurs on other nephric progenitor genes (*Pax8*, *Jag1* and *Lef1*), which is essential for differentiation of the metanephric mesenchyme into the appropriate nephric cell types [81].

Several histone methyltransferases (HMTs), including Ash21, Ezh2 and Suz12, have been associated with histone methylation events during embryonic kidney development. Ash21 facilitates H3K4 methylations, and Ezh2 and Suz12 facilitate the methylation of H3K9me2/3 and H3K27me3 [21]. Ash21 interacts with the Trithorax complex and induces the Pax transactivating domain-interaction protein (PTIP) pathway that regulates *Pax2* expression and, therefore, may be an effector of *Pax2*-dependent transcriptional regulation. Ezh2, a subunit of the Polycomb repressive complex 2 (PRC2), is purported to play a role in maintaining *Six2* expression in the early metanephric mesenchyme [21], and it regulates PRC2 expression in the cap mesenchyme [82]. Suz12, another subunit of PRC2, is highly expressed in the cap mesenchyme and in early nephron formation stages, similarly to Ezh2 [82]. G9a regulates the methylation of H3K9me2, which is found in *Pax2*-expressing cells in the maturing cap mesenchyme as well as distal segment of the S-shaped bodies [83]. *Dot1* only catalyzes the methylation of H3K79, which is increasingly expressed postnatally, suggesting a role of H3K79 methylation in postnatal maturation [84]. *Suv39h* regulates the methylation of H3K9me3 and plays an important role in overall embryonic development and genome stability [85].

Multiple Set1-like complexes, including human SET1 (hSet1), mixed-lineage leukemia 1 (MLL, MLL1, HRX, ALL1), mixed-lineage leukemia 2 (MLL2), mixed-lineage leukemia 3 (MLL3) and mixed-lineage leukemia 4 (MLL4, ALR), carry methyltransferase activities [80]. PTIP, a component of the breast cancer type 1 C Terminus (BRCT) domain, interacts with MLL3 and ALR as part of a histone methyltransferase complex to bind *Pax2*-dependent targets. This is known as the PTIP–MLL H3K4 methyltransferase complex, and it plays an important role in the differentiation of the metanephros mesenchyme from the intermediate mesoderm [86]. In addition, several known histone demethylases, including *Jmjd3* and *Utx*, which are involved in kidney development through catalyzing the demethylation of H3K27 [21]. *Jmjd3* expression decreases postnatally [87], and *Utx* expression increases postnatally [88], contributing to the steady levels of H3K27me3 throughout embryonic and kidney development.

Histone arginine methylations also may have a role in the regulation of gene expression in renal progenitor cells. H3R2me2 and H3R17me2 markers are highly expressed in both the cap mesenchyme and immature nephrons, whereas the H3R8me2 marker is mainly expressed in immature nephrons. All three arginine methylation markers are present in the maturing collecting ducts [21]. Further research is needed to better understand the role of arginine methylations in gene expression in kidney development.

Histone deacetylases (HDAC) are also described as playing an important role in the expression of the main renal progenitor genes [89]. HDAC1 and HDAC2 are expressed in the metanephric mesenchyme and progenitor cells, such as the comma and S-shaped bodies and the ureteric bud branches, and HDAC3 is abundantly expressed in the glomerular podocytes [89]. HDAC activity has been found to regulate the expression of *Osr1*, *Lhx1*, *Eya1*, *Pax2/8*, *WT1*, *Gdnf*, *Wnt4/9b* and other important nephrogenic genes [89]. *Pax2*, *WT1, Lhx1* and *Wnt4* were found to be downregulated in the absence of HDAC1 and HDAC2, whereas *Pax8* is still expressed in the early kidney mesenchyme. The ureteric bud branching genes *Foxd1* and *Bmp4* do not appear affected by the loss of HDAC activity, but *Spry1* and *Wnt9b* are suppressed without HDAC. Site-specific HDAC activity is yet to be fully elucidated, but it has become abundantly clear that HDAC activity is vital to the proper expression of renal progenitor genes and, subsequently, proper kidney development.

The major genes, their expression sites and roles in the developing metanephric kidney and the association of those genes with epigenetic regulators and markers are summarized in Table 1. The genes are divided into spatial groups, from the mesonephric and early metanephric development period (*Osr1*, *Lhx1* and *Pax2/8*), the metanephric development period (*Wt1, Foxd1*, *Hox11*, *Eya1*, *Six1/2*, *Sall1*, *Wnt9b* and *Gdnf*) and the nephron patterning and formation period (*Wnt4*, *Fgf8*, *Bmp7*, *Notch2*, *Tcf21/Pod*, *VEGF* and *Jag1*). A recent review of the current progress on epigenetics research in kidney development provides additional insights on the presented information [90].

## 7. The Application of Single-Cell Sequencing Techniques in Studying Kidney Development

Single-cell sequencing technologies can be used to detect the genome, transcriptome and other multi-omics of individual cells in specific organs, such as the kidney, which can reveal cell population differences and cellular evolutionary relationships. Compared with traditional sequencing technologies, which can only get the average of many cells, are unable to analyze a small number of cells and lose cellular heterogeneity information, single-cell technologies have the advantages of detecting heterogeneity among individual cells, distinguishing a small number of cells and delineating cell maps of specific organs [91]. Nowadays, single-cell sequencing technology is increasingly used in various fields. In this section, the recent progression of using single-cell sequencing methods in the study of kidney development is described, and the potential joint use of single-cell sequencing technologies in understanding epigenetic mechanisms in kidney development is discussed.

Single-cell RNA sequencing (scRNA-seq) has become one of the most useful tools for studying organ development, which can identify all RNA transcripts, coding and non-coding, in individual cells [92]. Single-cell transcriptomic analysis in kidneys can generate new information, including (1) redefining and identifying novel renal cell types based on global transcriptome patterns [93]; (2) identifying molecular mechanisms of kidney diseases, not only by temporal (acute or chronic) and target (glomerular or tubular) characteristics, but also by novel cell-type specific changes [94]; (3) reevaluating the accepted idea that plasticity only occurs in immature or nascent cells [95] and (4) identifying the readout of specific gene expression profiles in each renal cell type [96].

Because the developmental kidney contains progenitors and differentiated cells, as well as cells at intermediate developmental stages, it precludes the use of conventional high-throughput gene expression techniques. The use of scRNA-seq is still in its infancy. A scRNA-seq analysis has been performed on three different stages of developing mouse kidneys, including kidneys collected from embryonic days 15.5 and 17.5 and postnatal day 0 [97]. This study evaluated the expression of genes associated with various differentiation events at these three stages of kidney development, which provided a resource of single-cell transcriptomic profiles in embryonic kidneys for further investigation. In this study, 45 clusters of cells were identified and characterized using cell-type specific markers. Across the three stages studied, temporal shifts in expression of the marker genes and transcription factors were found to occur in individual nephron segments. Among the transcription factors identified in this study, *Bmp7* was found to be expressed across all stages, *Fgf8* was only found in the immature distal tubules and *Bmp4* was found in mature distal tubules. Most regulatory genes such as *Wnt*, *Fgf* and *Notch* were downregulated in the more mature stages, suggesting that activation and suppression of the renal progenitor genes is essential to the proper development of the kidney (see Figure 2). This study also characterized the stage-dependent expression of representative genes of different renal structures. For example, in podocytes, the expression of *Collagen type 4 alpha3 chain (Col4a3)*, *Semaphorin 3g (Sema3g)*, *High-temperature requirement A serine peptidase 1 (Htra1)* and *chloride intracellular channel protein 3 (Clic3)* is increased by P0, but it is still significantly decreased at E15.5. In the proximal tubules, expression of *solute carrier family 5 member 12 (Slc5a12)*, *Cytochrome P450 family 27b1 (Cyp27b1)* and *Keratin Associated Protein (Kap)* is increased by P0, but it is significantly decreased at E15.5. In the loops of Henle, the expression of *uromodulin (Umod)*, *prostaglandin E receptor 3 (Ptger3)* and *carbonic anhydrase 15 (Car15)* is stage-dependent, and in the collecting ducts, the expression of *aquaporin 2 (Aqp2)*, *cyclin-dependent kinase inhibitor 2b (Cdkn2b)* and *lipocalin 2 (Lcn2)* is also stage-dependent. Such characterizations could guide future investigation to determine how these regulatory and representative genes are regulated by epigenetic mechanisms in the different developmental stages.

Recent studies have revealed pervasive differences in renal embryogenesis between mice and humans. In order to shed light on human kidney development, single-cell RNA sequencing has been used to characterize gene expression in different cell populations and to study individual cell dynamics and lineage trajectories during human embryonic development. A single-cell transcriptomics study of the human fetal kidney identified 22 cell types and a host of marker genes in five different developmental ages of human fetal kidneys [98]. This study also identified several subpopulations of nephron progenitor cells (NPCs) that give rise to the nephron, the functional unit of the kidney. Kidney development requires the balance between two fundamental processes: growth and the creation of structure, which are reconciled by self-renewing progenitor cells. Understanding the genetic mechanisms regulating these processes is key for understanding kidney development. Another study analyzed human embryonic kidneys at weeks 9, 11, 13, 16 and 18 stages, and it identified four subtypes (described as NPCa-d) of nephrogenic progenitor cells differentiated by the levels of expressions of several key regulatory genes [99]. *SIX2*, *CITED1* and *EYA1*, along with *mesenchyme homeobox 1 (MEOX1),* are expressed in all nephrogenic progenitor cells but in varying levels across the four subtypes. Among four clusters of NPCs, NPCa is most likely the self-renewing compartment, which expressed the highest levels of *CITED1* and *TMEM100,* compared with the other NPCs. NPCb expresses several genes that modulate NOTCH, BMP and TGF-β pathway activity and low levels of *lymphoid enhancer binding factor 1 (LEF1)*. NPCc is distinguished from the other NPCs by higher expression of genes involved in or regulated by retinoic acid signaling, such as cellular retinoic acid binding protein 2 (*CRABP2*). NPCd expresses low levels of *OSR1*, *CITED1* and *MEOX1* and a higher level of *LEF1*. This study further indicates a developmental flow from NPCa via NPCb–c to NPCd and that NPCa gives rise to NPCb and NPCc, and NPCd may be a more proliferative intermediary state between the other nephron progenitor cell types and the pretubular aggregates that later become the renal vesicles. Based on the characterizations of the four subtypes, NPCa and NPCc may be located closer to the ureteric bud than NPCb and NPCd, which is consistent with the finding that NPCd is an intermediary state during the formation of the renal vesicles. In particular, NPCa seemed to be located closest to the tip of the ureteric bud, NPCb and NPCc were located closer to the ureteric bud stalk and NPCd was closest to the pretubular aggregates. These findings are consistent with reports in the literature of NPCs streaming from their niche at the ureteric bud tip towards the ureteric bud branch point to form the pretubular aggregate and, subsequently, the renal vesicle. A third study also demonstrated the heterogeneity of renal progenitor populations [100]. This study identified two subtypes within the cap mesenchyme, one with self-renewal potential and the other showing epithelial features through the mesenchymal to epithelial transition. The developmental profiles of these two cell types may explain how the cap mesenchyme sustains its progenitor state on the one hand, whereas on the other hand, it gives rise to the formation of nephron tubules. In order to better understand the mechanisms regulating nephron tubule segmentation, this study also examined lineages for the proximal tubules, loops of Henle and the collecting ducts during kidney morphogenesis, which laid a foundation for identifying key genetic markers during kidney development in order to better understand and treat renal diseases. Last, a study compared mouse and human embryonic kidney development and characterized a similar set of nephron progenitor cell subtypes in human and mouse kidneys [101], which further highlighted the importance of the emerging scRNA-seq technology as a way to overcome limitations of genetic approaches for studying human kidney development. The data produced by scRNA-seq analysis are compelling towards studying epigenetic mechanisms in the expression of these progenitor and regulatory genes and, therefore, the role of epigenetics in the complex process of kidney development.

Additionally, the application of single-cell transcriptomics to adult mouse kidneys also led to several important discoveries. One such study identified three subclusters of collecting duct cells: intercalated cells (IC), principal cells (PC) and transitionary cells [96]. The transitionary cell may represent a transition stage between PC and IC cells, suggesting that PC and IC cells may still undergo transitionary changes in both the immature and developed collecting ducts. *Notch* signaling is involved in the regulation of the transition of IC to PC cells in the adult collecting ducts, and dysregulation of this transition may result in chronic kidney disease (CKD) and metabolic acidosis. Furthermore, using known disease markers, this study revealed that kidney diseases generally show cell-type specificity and are limited to only one cell type. For example, proteinuria only involves the glomerular podocytes, renal tubule acidosis (RTA) only involves the IC cells of the collecting ducts, blood pressure dysregulation involves the distal convoluted tubules, nephrolithiasis only involves the proximal tubules, and CKD only involves the proximal tubules, which highlights the critical roles of each renal cell type in proper kidney function. In summary, scRNA-seq analysis lays the foundation for future research on understanding kidney development and may contribute to the further understanding of the progression of kidney diseases.

In addition to scRNA-seq analysis, the increasing interest in the epigenetics in kidney development is driving us to consider the application of experimental approaches for directly characterizing epigenomes at single-cell resolution. Methodologies for single-cell epigenomics include single-cell DNA methylome sequencing, single-cell ChIP-sequencing single-cell assay for transposase-accessible chromatin with sequencing (scATAC-seq) and single-cell Hi-C analysis.

Single-cell DNA methylome sequencing quantifies DNA methylation. This method is similar to single-cell genome sequencing but with the addition of a bisulfite treatment before sequencing [102]. Sequencing 5mC in individual cells can reveal how epigenetic changes across genetically identical cells from a single tissue or population give rise to cells with different phenotypes. Single-cell DNA methylome sequencing can also be used as scRNA-seq analysis to identify distinct cell types in kidneys. Potentially, this method can be applied to study the entire epigenome of complex cell populations at single-cell resolution. However, because of the high sequencing burden, the scaling of high depth single-cell bisulfite sequencing to many single cells is still limited, which may be improved through the combination with techniques for targeted enrichment and an alternative experimental design to lower sequencing depth [103].

Single-cell ChIP-sequencing is a method used to analyze protein interactions with DNA at single-cell resolution. Single-cell ChIP-seq is extremely challenging due to background noise caused by nonspecific antibody pull-down. A study with this method so far has been performed successfully to study chromatin states in breast cancer [104]. Single-cell chromatin mapping to reduce the level of background noise in chromatin mapping is also an important avenue for the further development of single-cell chromatin-mapping methods.

Single-cell assay for transposase-accessible chromatin with sequencing (scATAC-seq) maps chromatin accessibility across the genome. In this method, a transposase inserts sequencing adapters directly into open regions of chromatin, allowing those regions to be amplified and sequenced [105]. scATAC-seq is able to separate cells based on their cell types, uncover sources of cell-to-cell variability, and show a link between chromatin organization and cell-to-cell variation. scATAC-seq has been used in combination with scRNA-seq to evaluate the effect of chromatin interactions with gene expression [106].

Chromosome conformation capture techniques (often abbreviated to 3C technologies) are a set of molecular biology methods used to analyze the spatial organization of chromatin in a cell [107]. Single-cell Hi-C is a modification of the original Hi-C protocol, which is an adaptation of the 3C method, that allows the proximity of different regions of the genome to be determined and three dimensional maps of entire genomes in a single cell to be generated [108]. This method was made possible by performing the digestion and ligation steps in individual nuclei, as opposed to the original Hi-C protocol, where ligation was performed after cell lysis in a pool containing crosslinked chromatin complexes. In single cell Hi-C, after ligation, single cells are isolated, and the remaining steps are performed in separate compartments, and hybrid DNA is tagged with a compartment-specific barcode. After that, high-throughput sequencing is then performed on the pool of the hybrid DNA from the single cells.

The potential application of single-cell epigenomics methods in kidney development and diseases include: (1) to characterize epigenomics in small cell niches in the kidneys; (2) to reconstruct the distribution of epigenomic states within mixed cell populations, which may result in the classification of single cells into known types or the identification of novel subpopulations with distinct epigenomes in kidneys; (3) to allow inference of the long-range correlations of epigenetic mechanisms and dynamics of epigenetic information within cell populations, and thereby contribute to the mechanistic understanding of epigenetic reading, writing and maintenance in kidneys; (4) to allow the integration with single-cell RNA-seq data in kidneys. Single-cell epigenomics can be enhanced by the available scRNA-seq transcriptional data generated from embryonic and adult kidneys. This will allow for computational integration of the data into a model that infers epigenomic and transcriptional subpopulations with compatible frequencies. Such subpopulations can then be explored to detect correlation between gene regulation and epigenetic mechanisms in kidney development, which should lead to unexpected discoveries. As single-cell sequencing technologies continue to improve, the ability to combine different single-cell sequencing techniques should lead to the concept of multi-omics. We believe that single-cell epigenomics will become an essential tool in epigenetics and genome-regulation research, as it naturally fills a historical gap between traditional microscopic examination of epigenetic processes and modern (bulk) genomics.

## 8. Conclusions and Perspectives

Kidney development involves various cellular components and requires multiple signaling pathways working together to induce proper formation of the ureteric bud, branching of the ureteric bud, the formation of the renal vesicle and the nascent nephron and the maturation of all kidney structures. Major genetic markers and signaling pathways in kidney development have been identified. Recent studies are focusing on identification of epigenetic markers during kidney development and understanding of epigenetic mechanisms in the regulation of signaling pathways associated with kidney development, which can be facilitated by the development of single-cell sequencing techniques. Many of the pathways critical for kidney development also contribute to regeneration of renal cells or to the initiation or progression of renal disease. In the future, single-cell sequencing technologies may be integrated with multi-omics methods to investigate kidney development and to understand epigenetic mechanisms in kidney disease progression. The efforts to simplify the protocols and further reduce the detection cost of single-cell sequencing technologies are necessary so that the technologies can be applied to basic research of kidney development, and they should also play an important role in clinical diagnosis and treatment of kidney diseases.

## Figures and Tables

**Table 1:** Important genetic factors regulating proper kidney development and their associated epigenetic regulators and markers.

Gene	Expression	Role(s)	Epigenetic Regulators and Markers
**Mesonephric and early metanephric development**			
*Osr1*	LPM, IM	Regulate development of posterior nephric structures	H2A.Z, HDAC, Polycomb/Trithorax
*Lhx1*	LPM, ND	Regulate development of the metanephric duct and continued renal development	H3K9me2 and H3K27me3, HDAC
*Pax2*	IM, ND	Regulate branching of the ureteric bud and continued renal development	H3K4 methyltransferase complex, H3K9me2 and H3K27me3, HDAC, Polycomb/Trithorax (Ash21)
*Pax8*	IM	Regulate branching of the ureteric bud and continued renal development	H3K9me2 and H3K27me3, HDAC
***Metanephric development***			
*Wt1*	IM, MM	Regulates continued differentiation of metanephric progenitor cells	HDAC
*Foxd1*	MM, SC	Regulates nephron endowment and continued branching of the ureteric bud	HDAC
*Hox11*	MM	Regulates development of the metanephros	Polycomb/Trithorax
*Eya1*	MM	Regulates initiation of mesoderm differentiation and formation of the initial ureteric bud	HDAC
*Six1*	MM	Regulates formation of the initial ureteric bud and subsequent branching of the ureteric bud	
*Six2*	MM, CM	Regulates formation of metanephric caps and subsequent nephron formation	H3K9me2 and H3K27me3, Polycomb/Trithorax (Ezh2), G9a
*Sall1*	MM	Regulates branching of the ureteric bud and formation of new nephrons	Polycomb/Trithorax
*Wnt9b*	UB	Regulates differentiation of metanephric caps and subsequent formation of new nephrons	HDAC
*Gdnf*	MM	Regulates the formation and branching of the ureteric bud	HDAC, Ret
***Nephron patterning***			
*Wnt4*	CM	Regulates metanephric cap behavior and subsequent nephron formation	HDAC
*Fgf8*	MM, CM	Regulates continued nephron formation and proper renal development	
*Bmp7*	UB, MM	Regulates continued branching of the ureteric bud and nephron endowment	
*Notch2*	RV, SB	Regulates proper development of proximal tubules of nephrons	HDAC
*Tcf21 (Pod1)*	SC, PC	Regulates differentiation of podocytes	
*Pdgfr*	PC	Regulates development of the glomerulus	
*VEGF*	GP	Regulates development and survival of the glomerulus	
*Jag1*	GP, ND	Regulates notch signaling pathways	H3K9me2 and H3K27me3, H3K4me3

CM, cap mesenchyme; IM, intermediate mesoderm; LPM, lateral plate mesoderm; MM, metanephric mesenchyme; ND, nephric duct; PC, podocyte cells; RV, renal vesicles; SB, S-shaped body; SC, stromal cells; UB, ureteric bud; GP, glomerular podocytes.
